# Tagging of the vaccinia virus protein F13 with mCherry causes aberrant virion morphogenesis

**DOI:** 10.1099/jgv.0.000917

**Published:** 2017-09-20

**Authors:** David C. J. Carpentier, Michael S. Hollinshead, Helen A. Ewles, Stacey-Ann Lee, Geoffrey L. Smith

**Affiliations:** Department of Pathology, University of Cambridge, Tennis Court Road, Cambridge CB2 1QP, UK; ^†^​ Present address: The Francis Crick Institute, 1 Midland Road, London NW1 1AT, UK.

**Keywords:** *Poxviridae*, Vaccinia, F13, A36, F12, virus morphogenesis, virus wrapping, IEV, electron microscopy, virus egress, fluorescent protein tagging

## Abstract

Vaccinia virus produces two distinct infectious virions; the single-enveloped intracellular mature virus (IMV), which remains in the cell until cell lysis, and the double-enveloped extracellular enveloped virus (EEV), which mediates virus spread. The latter is derived from a triple-enveloped intracellular enveloped virus (IEV) precursor, which is transported to the cell periphery by the kinesin-1 motor complex. This transport involves the viral protein A36 as well as F12 and E2. A36 is an integral membrane protein associated with the outer virus envelope and is the only known direct link between virion and kinesin-1 complex. Yet in the absence of A36 virion egress still occurs on microtubules, albeit at reduced efficiency. In this paper double-fluorescent labelling of the capsid protein A5 and outer-envelope protein F13 was exploited to visualize IEV transport by live-cell imaging in the absence of either A36 or F12. During the generation of recombinant viruses expressing both A5-GFP and F13-mCherry a plaque size defect was identified that was particularly severe in viruses lacking A36. Electron microscopy showed that this phenotype was caused by abnormal wrapping of IMV to form IEV, and this resulted in reduced virus egress to the cell surface. The aberrant wrapping phenotype suggests that the fluorescent fusion protein interferes with an interaction of F13 with the IMV surface that is required for tight association between IMVs and wrapping membranes. The severity of this defect suggests that these viruses are imperfect tools for characterizing virus egress.

## Abbreviations

CEV, cell-associated enveloped virus; EcoGPT, E. coli guanine phosphoribosyltransferase; EEV, extracellular enveloped virus; GmC, A5GFP-F13mCherry; IEV, intracellular enveloped virus; IMV, intracellular mature virus; KLC, kinesin light chain; mC, mCherry; VACV, vaccinia virus; WT, wild-type.

## Introduction

The family *Poxviridae* comprises large DNA viruses that replicate in the cytoplasm of their host cell [1]. Poxvirus replication has been studied most extensively using vaccinia virus (VACV), the prototype member of the family and the live vaccine used in the eradication of smallpox [2]. During VACV replication infectious virions known as intracellular mature virus (IMV) are formed within virus factories [3]. Some IMVs migrate from the viral factories in a microtubule (MT)-dependent manner [4, 5] and are wrapped by a double envelope derived from trans-Golgi [6] or early endosomal [7] membranes. These virions, known as intracellular enveloped viruses (IEVs), are transported towards the cell surface (for a review see [8]) along MTs in a kinesin-1-dependent manner [9–12], where their outer envelope fuses with the cell membrane. Virions are either released as extracellular enveloped virions (EEVs) or remain attached to the cell surface as cell-associated enveloped virions (CEV). Nucleation of actin polymerization underneath CEVs results in the formation of actin tails projecting virions away from the cell (reviewed in [13]).

Three VACV proteins, A36, F12 and E2, influence the transport of IEVs by kinesin-1. Viruses lacking A36 [14], F12 [15, 16] or E2 [17] form IEV normally [18], but have defects in IEV transport to the cell surface. A36 is an integral membrane protein that is enriched in the outer envelope of IEVs [19] and associates with the kinesin light chain (KLC) component of kinesin-1 [20, 21]. Recently, A36 was shown to bind specifically to KLC isoform 1 and not to KLC2 [22]. Intracellular movement of IEVs at speeds consistent with MT-mediated trafficking occurs in the absence of A36, albeit at reduced efficiency and with reduced run lengths, and surface virions are present at 50 % of wild-type (WT) levels late during infection [23]. In addition, A36 is required for F12 association with IEVs [24].

F12 and E2 are cytoplasmic proteins that form a complex associated with IEVs during egress [25]. The F12/E2 complex associates preferentially with KLC2 via an interaction between E2 and the KLC C-terminal tail [26]. Viruses lacking either F12 or E2 display similar phenotypes, resulting in almost complete loss of IEV egress [18], and the association of either protein with IEVs is dependent on the other [25].

To study the roles of A36 and the F12/E2 complex in virion trafficking in greater detail we sought to generate fluorescently-labelled viruses that would enable the comparison of IEV movement in different mutant viruses by live-cell microscopy. Several viruses containing fluorescent proteins fused to virion proteins have been reported. Green fluorescent protein (GFP) fused to capsid protein A5 (product of the VACVWR123 open reading frame) was used to visualize virion movement along MTs following entry [27]. This A5GFP labels all virions (naked cores, IMVs, IEVs, CEVs and EEVs) in infected cells. To track the movement of IEVs and differentiate them from IMVs, several laboratories have fused fluorescent proteins to either F13 [9, 28] or B5 [10, 29] that are present in the IEV, CEV and EEV but are absent from IMV. B5 is a type I transmembrane glycoprotein with the majority of the protein projecting into the lumen of the wrapping membranes and exposed on the surface of the CEV/EEV [30, 31]. F13 is a palmitoylated protein that is associated with the cytoplasmic side of membrane-bound vesicles [32, 33] and is present between the IMV and EEV membranes of IEV and CEV/EEV. B5 and F13 are both required for efficient IEV wrapping [34–36]. However, they also associate with cellular vesicles during their transport to the site of wrapping. B5 and F13 take different routes to the site of wrapping [37]. For B5 this involves exocytosis to the cell surface followed by recycling by endocytosis [38, 39].

To quantify and compare the movement of IEV of different mutant viruses, we generated recombinant viruses in which both capsids and IEV membranes were labelled with fluorescent proteins. No detrimental effect of tagging F13 with GFP to its C-terminus has been reported [9, 28]. Considering that a double fluorescent virus expressing mCherry-tagged A5 and GFP-tagged F13 has been described and used to characterize the steps involved in EEV entry [40], a similar strategy was used here to introduce F13 fused to mCherry (F13mC) into viruses already expressing A5GFP. This study reports that viruses expressing F13mC undergo aberrant wrapping that results in reduced virus egress and spread, which are particularly evident in viruses lacking A36.

## Results

### Imaging of two-colour fluorescent IEVs using a transfection/infection protocol

**Fig. 1.**
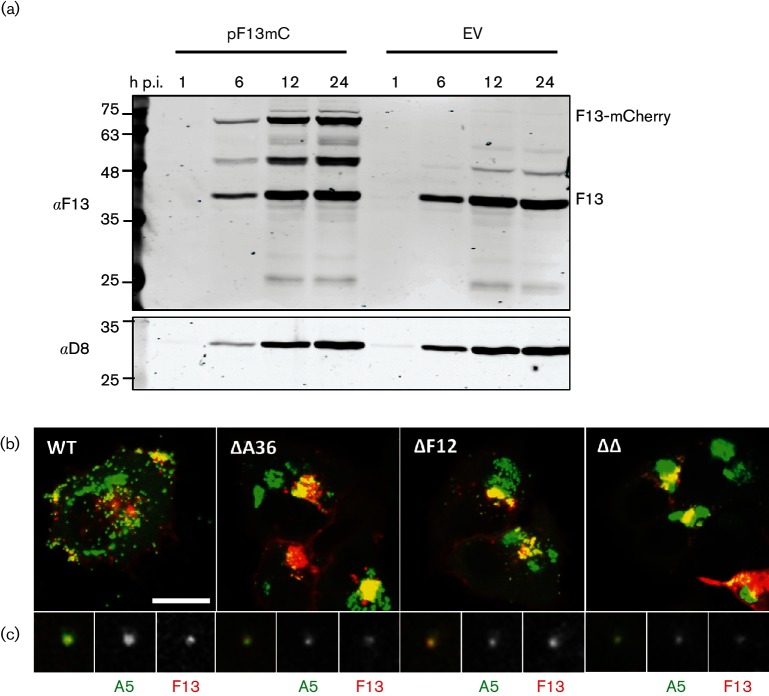
Imaging A5GFP- and F13mCherry-positive IEVs following transfection/infection. (a) F13 and F13mCherry expression in HeLa cells. Cells were transfected with pF13mC or pSJH7 (EV) and 24 h later infected with vA5GFP at 5 p.f.u./cell. Cell lysates were prepared at the indicated time (h) p.i. and analysed by SDS-PAGE and immunoblotting for VACV proteins F13 or D8. The positions of the molecular mass markers are indicated in kDa on the left. (b) Still images taken from movies of live HeLa cells transfected with pF13mC (red) and infected with the indicated A5GFP (green)-expressing viruses, recorded at 8–10 h p.i. The scale bar represents 20 µm. (c) High-magnification view of a single virion positive for both A5GFP and F13mCherry.

To generate fluorescent IEVs, F13 was tagged at its C terminus with the mCherry fluorescent protein (F13mC) using the same strategy as described for GFP [28]. A plasmid was constructed carrying the *F13L* orf fused to the mCherry gene, flanked by 350 bp of both upstream (including the F13 promoter region) and downstream sequence from the VACV WR *F13L* locus. This plasmid [or an empty vector (EV) control] was transfected into cells for 12 h and subsequently cells were infected with VACV expressing A5GFP (vA5GFP) [41]. The expression of F13mC from this plasmid (pF13mC) and WT F13 from vA5GFP were analysed at different times post-infection (p.i.) by SDS-PAGE and immunoblotting (Fig. 1a). F13 (37 kDa) is a late protein [32] and was only detected from 6 h p.i. onwards. F13mC (~66 kDa) was expressed with similar kinetics and at similar levels to WT F13, suggesting that this transfection/infection approach did not result in over-expression of F13mC.

Expression of F13mC in cells infected with vA5GFP should result in the formation of double-labelled IEVs, with A5GFP in the core of all virions and F13mC in the outer membranes of IEVs, enabling these virions to be analysed by live-cell microscopy. To test this, HeLa cells were transfected with pF13mC and infected with vA5GFP and imaged between 8 and 10 h p.i. (see Videos S1–S4, available in the online Supplementary Material). After optimization of acquisition settings, each cell could be imaged for a total of 20 min before photobleaching of the mCherry signal. GFP+ve mCherry –ve IMVs, GFP+ve mCherry+ve IEVs and GFP-ve mCherry+ve subcellular vesicles could all be seen moving around the infected cell. Several IEVs trafficking from the site of wrapping to the cell periphery could be tracked. The imaging was repeated using A5GFP viruses defective for the expression of A36 (v∆A36-A5GFP), F12 (v∆F12-A5GFP) [23] or both (v∆A36∆F12-A5GFP) [42]. In all samples, viral factories (Fig. 1b, perinuclear green structures) and sites of wrapping (Fig. 1b, yellow structures located near to the nucleus) were clearly identifiable, and double-fluorescent virions could be imaged (Fig. 1c). In vA5GFP-infected cells these virions were clearly motile, but in cells infected with viruses lacking either F12 and/or A36, motile IEVs were either absent or much reduced. Although immunoblotting showed comparable levels of F13 and F13mC during infection (Fig. 1a), imaging of individual cells showed highly variable levels of F13mC expression. Notably, cells expressing very high levels of F13mC displayed an aberrant morphology or showed evidence of membrane blebbing (for an example see the F13mC-expressing cell at the left-hand edge of Video S1). The low number of trackable virions, the heterogeneity of F13mC expression and the apparent toxicity of F13mC at high levels made this approach unsuitable for the quantitative comparison of IEV movement. Therefore, recombinant viruses expressing both A5GFP and F13mC from their endogenous viral promoters were constructed as an alternative approach.

### Construction and analysis of double-fluorescent recombinant viruses

The F13mC plasmid was used to rescue a virus lacking F13 (v∆F13) that is defective in virus spread and produces a very small plaque [36, 43]. Introducing F13mC into v∆F13 by homologous recombination resulted in a considerable increase in plaque size and such viruses expressing F13mC (vF13mC) were isolated easily.

**Fig. 2.**
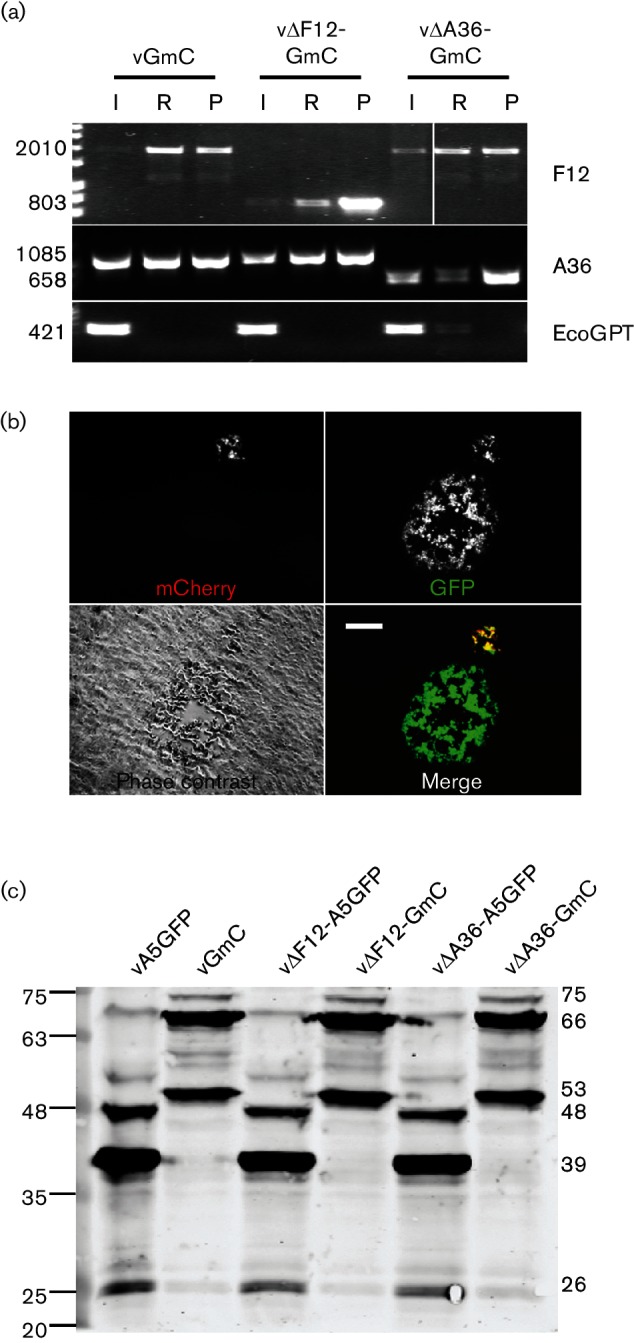
Generation and analysis of GmC viruses. (a) PCR analysis of the genomes of intermediate (I), parental (P) and recombinant VACVs. GFP+ve and mCherry+ve plaques were selected after three rounds of plaque purification in the absence of drug selection (R) and genomic DNA was analysed with primers specific for the A36, F12 and EcoGPT genes, as indicated. The sizes of the PCR products are indicated on the left in bp. (b) Epi-fluorescence microscopy showing two plaque phenotypes observed on BS-C-1 cells after passaging a v∆A36-GmC intermediate (EcoGPT+ve) virus through D98-OR cells in the presence of 6-thioguanine. Viruses expressing WT F13 produced large plaques, while viruses that expressed F13-mC produced a small plaque. Plaques were imaged at 4 days p.i. The scale bar indicates 250 µm. (c) Analysis of F13 and F13mC expression by the recombinant VACVs. BS-C-1 cells were infected with the indicated viruses at 10 p.f.u./cell for 16 h. Lysates of these cells were prepared and analysed by SDS-PAGE and immunoblotting with a mAb against VACV protein F13. The sizes of the F13 proteins are shown on the right and the positions of the molecular mass markers are indicated on the left (kDa). The calculated molecular mass (kDa) of the different F13 bands is indicated on the right.

Generating double-fluorescent viruses using pF13mC and the various A5GFP-expressing viruses would not result in plaque size rescue. Therefore transient dominant selection (see the Methods section) was used to introduce F13mC into vA5GFP, v∆A36-A5GFP and v∆F12-A5GFP. Two rounds of plaque purification in the presence of mycophenolic acid (MPA) of GFP+ve and mCherry +ve viruses resulted in intermediate EcoGPT-expressing viruses in which a single recombination event had inserted the whole F13mC plasmid into the F13 locus. All of these intermediates had similar plaque sizes to their parental virus. MPA selection was then removed and GFP+ve and mCherry+ve viruses produced using parent virus vA5GFP (to produce vGmC) or v∆F12-A5GFP (to produce v∆F12-GmC) yielded clones that had resolved and excised the EcoGPT cassette. However, the viruses generated from v∆A36-A5GFP did not resolve and had not lost the EcoGPT cassette (Fig. 2a), even after additional rounds of plaque purification. To force this resolution an EcoGPT+ve v∆A36 intermediate was passaged in D98-OR cells (HGPRT negative) in the presence of 6-thioguanine to inhibit the replication of EcoGPT-expressing viruses. When the resulting progeny were plated onto fresh cell monolayers, two distinct plaque size phenotypes were seen. Viruses expressing WT F13 produced plaques of similar size to the parental virus (Fig. 2b, large green plaque), while viruses that had resolved to express F13mC produced tiny plaques (Fig. 2b small yellow plaque).

Suspecting that a detrimental mutation had been introduced into this virus, the process was repeated by independent transfection/infection. However, this also yielded a tiny plaque phenotype that was GFP- and mC-positive, suggesting that F13mC was detrimental to virus spread in the absence of A36. To confirm that this defect was due to the mCherry tagging of F13, and not some other off-target mutation, a revertant virus was constructed by replacing the F13mC allele in v∆A36-GmC with a WT F13 allele to produce a v∆A36-A5GFP revertant. This rescued the plaque size back to that of the parental v∆A36-A5GFP.

The expression of F13 and F13mC in the different virus backgrounds was examined by immunoblotting with mAb against F13 and revealed three major bands (Fig. 2c). The most prevalent (~38 kDa) represented F13 [32, 44]. Minor bands were also present at 48 and 26 kDa. In viruses expressing F13mC, three bands were detected, each corresponding to one of the WT F13 bands with a ~28 kDa mCherry protein attached to it. While the relative abundance of the three bands differed slightly between WT F13 and F13mC, there was no difference in the levels of F13 or F13mC that could explain the large plaque size reduction in ∆A36 viruses.

### The plaque size defect is not limited to v∆A36

**Fig. 3.**
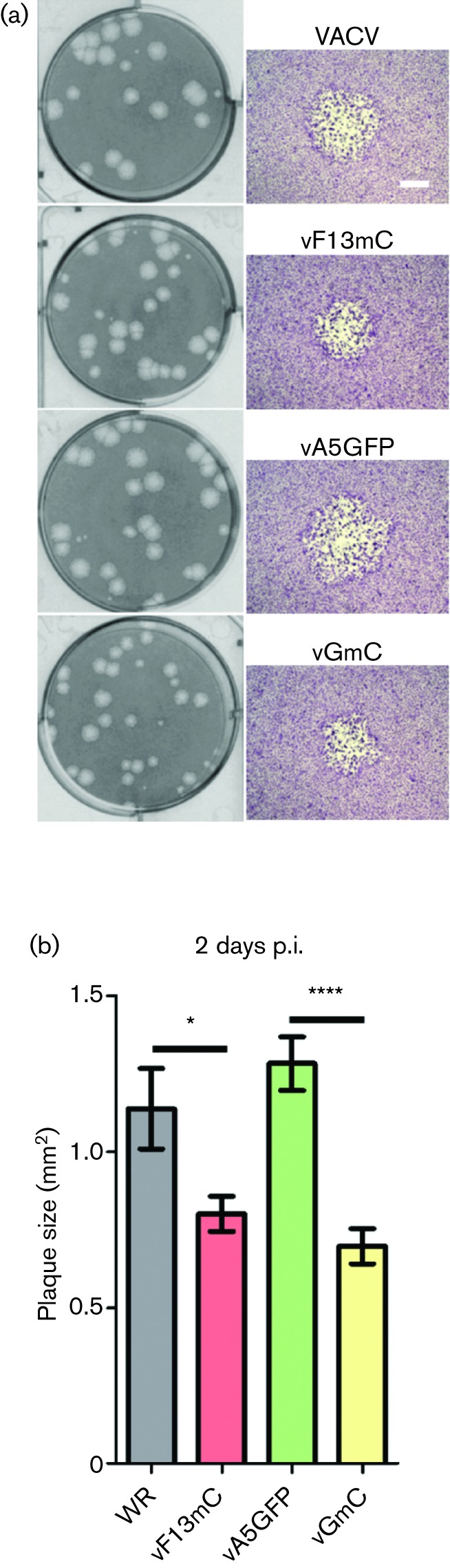
Plaque size analysis of recombinant viruses. (a) BS-C-1 cells were infected with the indicated viruses at 20–30 p.f.u./well and stained 5 days p.i. (left column) or 2 days p.i. (right column). At 2 days p.i. a representative plaque for each virus is shown (scale bar=500 µm). b) At 2 days p.i. 30 plaques for each virus from 3 different cell monolayers were photographed and the average area±sem was calculated. The plaque sizes of F13mC-expressing viruses were compared to those of their WT F13-expressing counterparts using Student's *t*-test (*, *P*<0.05; ****, *P*<0.0001).

**Fig. 4.**
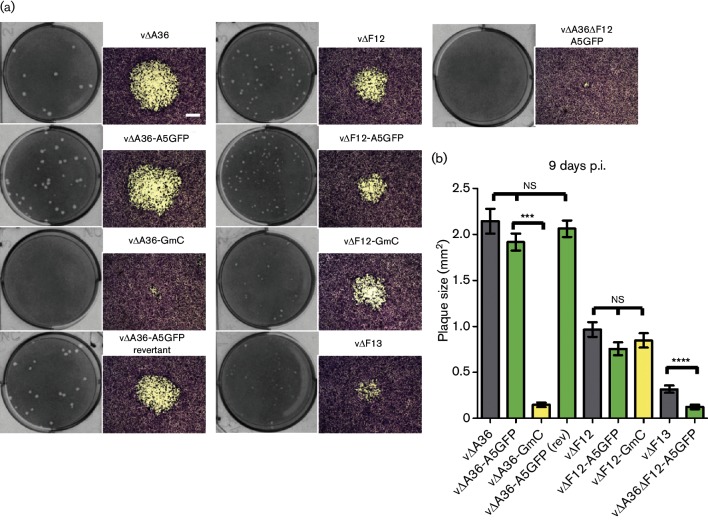
Plaque size analysis of recombinant viruses. (a) BS-C-1 cells were infected with the indicated viruses at 20–30 p.f.u./well and plaques were stained and photographed 9 days p.i. (scale bar=500 µm). (b) Plaque sizes were measured as described for Fig. 3b. The plaque sizes of viruses lacking A36 or F12 were compared using one-way ANOVA with Tukey’s multiple comparison test (ns, non-significant; ***, *P*<0.001; ****, *P*<0.0001). A virus lacking F13 expression (v∆F13) is included for comparison.

Viruses expressing F13 fused to GFP had not been reported to have a plaque size defect [9, 28]. Similarly, the viruses isolated here that expressed F13mC in the WT or ∆F12 backgrounds had no obvious plaque size defect compared to the parental virus. This suggested that only v∆A36-GmC had a spreading defect compared to its parent virus. A more thorough analysis of the plaque size of all the viruses was undertaken by measuring the average area of a large number of plaques from several independent infections. This analysis confirmed the large plaque size defect of v∆A36-GmC compared to both its parent and revertant viruses, but it also revealed that the plaque size defect was not limited to v∆A36-GmC. Both vF13mC and vGmC produced plaques that were slightly smaller than those of their respective parent viruses (Fig. 3). No significant plaque size defect could be detected for v∆F12-GmC (Fig. 4). It is interesting to note that the plaque size defect of v∆A36-GmC was even more severe than that for v∆F13. In fact, the plaque size was very similar to that produced by a virus lacking both A36 and F12 expression [42].

### The plaque size phenotype is due to reduction in virus egress

**Fig. 5.**
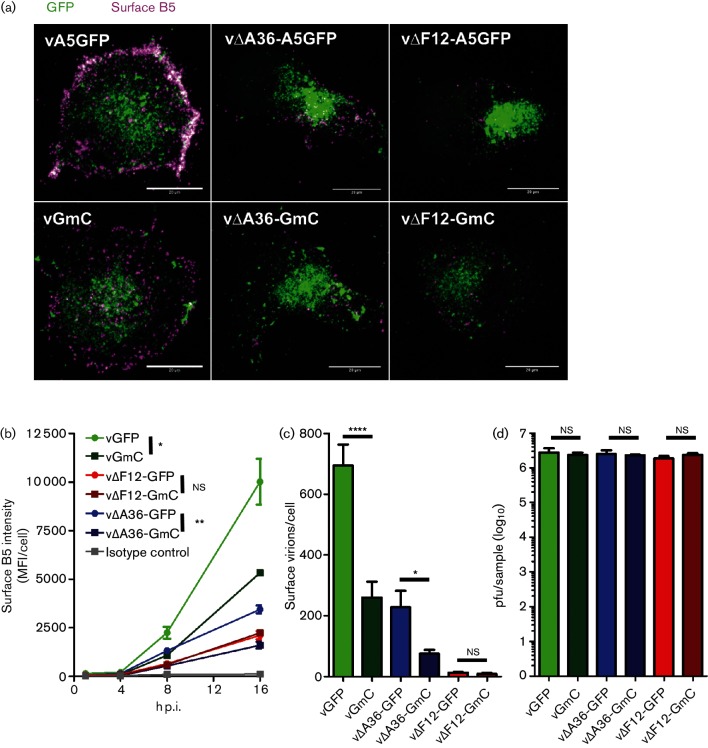
Egress assay comparison of recombinant viruses. (a) Confocal laser scanning microscopy of BS-C-1 cells infected with the indicated viruses. At 8 h p.i. live cells were stained with mAb for VACV protein B5 (red). The images are maximum-intensity projections of z-stack-acquired images encompassing the entire volume of the cell showing the A5GFP signal (green, virus capsids) and the surface B5 staining (magenta). Scale bars=20 µm. (b) Levels of B5 on the surface of BS-C-1 cells treated as for (a) at the indicated times p.i. and analysed by flow cytometry. The values shown are the mean fluorescence intensities (MFI) of a minimum of 2500 events. The isotype control represents vGmC-infected cells stained with a mAb to an intracellular VACV epitope not exposed on the CEV surface (see the Methods section). The graph shown is representative of three independent experiments. The egress levels at 16 h p.i. for each of the GmC viruses were compared to those of the A5GFP parental virus using Student's *t*-test (ns, non-significant; *, *P*<0.05; **, *P*<0.01). (c) BS-C-1 cells were infected and treated as in (a) and at 12 h p.i. the number of CEV particles (B5+ve, GFP+ve) on the cell surface were counted. The values shown are the averages of a minimum of 12 cells (4 each from 3 independently infected and stained coverslips) +sd and are representative of 2 independent experiments. The values for each GmC virus were compared to those of the A5GFP parental virus using Student's *t*-test (ns, non-significant; *, *P*<0.05; ****, *P*<0.0001). (d) Virus replication. BS-C-1 cells were infected at 10 p.f.u./cell and 18 h later the total virus yield was titrated by plaque assay on BS-C-1 cells. Triplicate infections were carried out for each virus. The average titre per sample is shown +sd. Each GmC sample was compared statistically to its A5GFP parent by a linked Student's *t*-test (ns, non-significant).

To determine whether the reduction in the plaque size of the F13mC-expressing viruses was due to reduced numbers of virions reaching the cell surface, the levels of B5 on the cell surface were measured after staining live infected cells with a mAb to protein B5 (Fig. 5a). As reported, viruses lacking A36 or F12 had reduced, or greatly reduced, surface B5. By comparison, B5 was also reduced from WT levels in vGmC-infected cells. Quantification by flow cytometry (Fig. 5b) and direct counting of B5 positive virions (CEVs) by confocal microscopy (Fig. 5c) confirmed these observations. Both vGmC and v∆A36-GmC showed lower surface B5 levels than their GFP parental viruses, each resulting in about a third of the number of virions reaching the cell surface compared to the parental virus. The surface B5 levels measured by flow cytometry for v∆F12-GmC did not differ from those of its parent GFP virus, and the slight reduction in the number of surface virions counted by confocal microscopy was not statistically significant (Student's *t*-test, *P*=0.17). Thus those viruses that showed a plaque size reduction also showed a defect in egress. These differences were not due to differences in virus replication, because the yields of infectious GmC viruses at 18 h p.i. were indistinguishable from those of their parental A5GFP viruses (Fig. 5d).

### Identification of a wrapping defect in F13mC-expressing viruses by electron microscopy

**Fig. 6.**
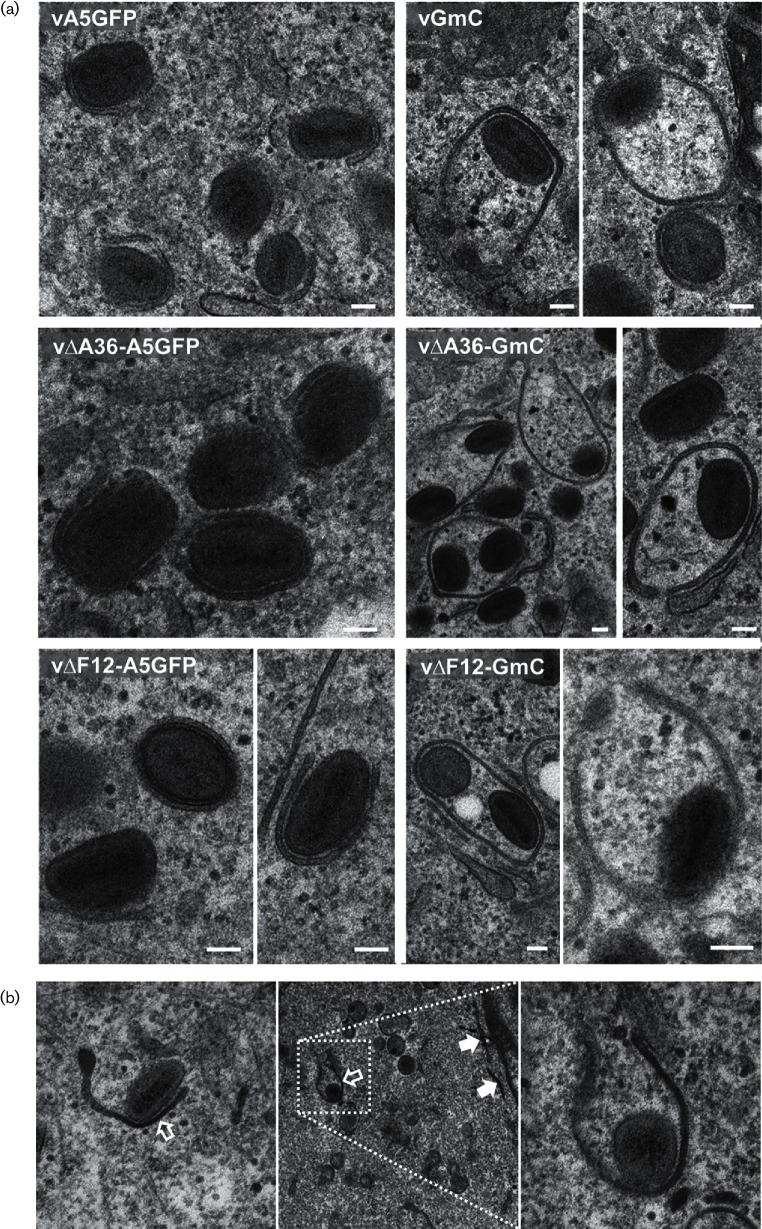
Analysis of virus wrapping by electron microscopy. (a) HeLa cells were infected with the indicated viruses at 5 p.f.u./cell and at 16 h p.i. were processed for transmission electron microscopy. Comparison of A5GFP viruses, where wrapping membranes and IMVs are tightly apposed, and GmC viruses, where wrapping membranes are less tightly associated with virions forming large enclosed bubble-like structures containing extra cytoplasmic material. Scale bars=100 nm. (b) Transmission electron microscopy of vGmC-infected cells treated with a liquid-phase marker for 60 min. The dark staining of the lumen between membranes (indicated by white arrows) associating with virions (open arrows) reflects their endosomal origin. Stained tubular endosomes are indicated by filled arrows in the middle panel. The panel on the right is a magnified view of the wrapping event shown in the middle panel.

**Fig. 7.**
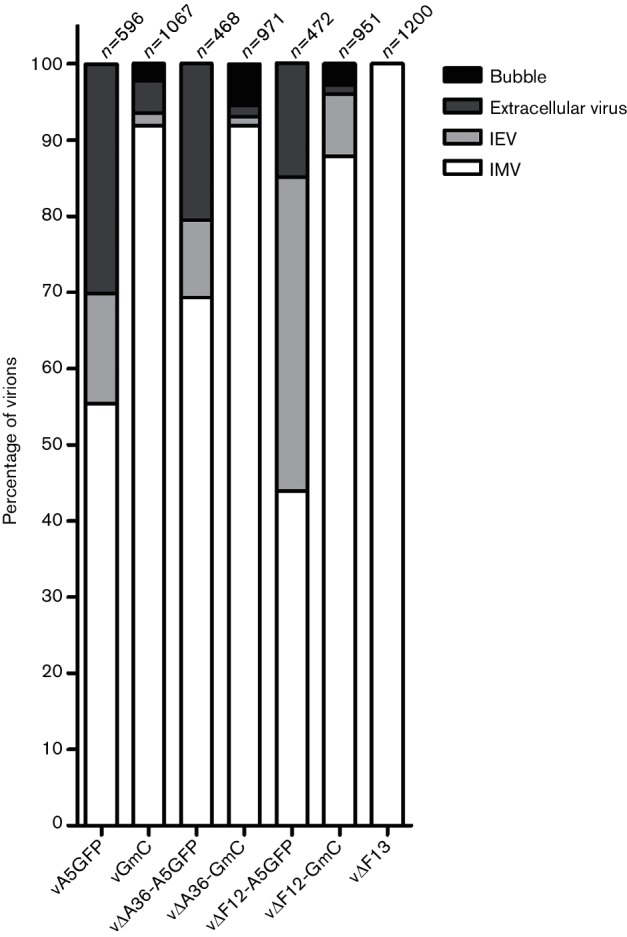
Quantitation of different virion types by electron microscopy. The different virion types identified in electron micrographs produced by the various A5GFP and GmC viruses were quantified. Between 472 and 1200 virions were counted for each virus from a minimum of 10 complete cell profiles per sample. The percentage of IMVs, IEVs (partially or completely wrapped) ‘bubbles’ (IMV enclosed within loosely associated wrapping membranes) and released extracellular virions is shown. v∆F13 is included as a control virus unable to form IEVs.

Fewer virions reaching the cell surface could indicate a defect in IEV formation or their transport. To investigate this, infected cells were studied by electron microscopy (Fig. 6). In the A5GFP viruses expressing WT F13, IMVs in the process of being wrapped with a double-membrane envelope, and fully wrapped IEVs were identified easily (Fig. 6a). In viruses expressing F13mC the wrapping process was abnormal. Some wrapping membranes failed to tightly surround virions, resulting in large ‘bubble’-like structures containing significant volumes of cytoplasmic material, and some of these ‘bubbles’ enclosed multiple virions. Analysis of samples that had been treated with a fluid-phase marker prior to fixation showed that the membranes forming these bubbles were of endosomal origin (Fig. 6b), indicating they are derived from the same membrane pool as those that form the normal IEV envelopes [7]. These aberrant wrapping structures were found in all the F13mC-expressing viruses, vGmC, v∆A36-GmC and v∆F12-GmC. The relative abundance of different virion types was quantified by counting the IMVs (including partially wrapped IMVs), fully formed IEVs, aberrantly wrapped bubbles and extracellular virions (including those associated with actin tails) present in a minimum of 10 cell profiles per sample (Fig. 7). Viruses expressing F13mC had more IMVs than enveloped virion types, indicating a bottleneck at the point of wrapping. Additionally, some of the wrapped virions were aberrant, forming bubbles. This wrapping defect was completely absent from viruses expressing WT F13. Interestingly, v∆A36-GmC produced a greater proportion of these bubble virions (compared to total virions) than either vGmC or v∆F12-GmC (Fig. 7).

## Discussion

This study describes the characterization of a panel of double-fluorescent VACVs that were constructed to enable measurement of IEV movement in cells infected with viruses lacking either A36 or F12, proteins that are involved in kinesin-1-mediated egress of IEV. A36 is the only protein known to link IEVs directly to the kinesin-1 complex, but in its absence IEV egress on MTs still occurs, albeit at reduced efficiency [23]. In the absence of a functional F12/E2 complex, A36 is unable to contribute to virus egress [42]. The F12/E2 complex can also bind kinesin-1 via the C-terminal region of KLC2 [26], but F12 and E2 are not integral membrane proteins, and the association of F12 with IEVs was reported to be dependent on A36 [24]. Therefore, there must be an alternative link between IEVs and kinesin-1 that can mediate IEV egress in the absence of A36. The IEV envelope incorporates several cellular proteins [45, 46] and it is possible that one of these forms the IEV/kinesin-1 link. Alternatively, one of the other VACV IEV membrane-associated viral proteins, A33, A34, B5, A56 or F13, might also do so. Most of these only have a small cytoplasmic domain, with the majority of the polypeptide being oriented into the membrane lumen (for a review see [47]), and a yeast-2-hybrid screen [21] and co-immunoprecipitation screen [22] failed to identify a direct interaction between any of these proteins and KLCs.

To characterize IEV egress and compare the type of virion movement that takes place when various components of the virus trafficking complex are absent, a panel of double-fluorescent viruses was generated to enable differentiation of IEVs from other virion types in infected cells. During the construction of viruses expressing F13 tagged with mCherry, a plaque size defect was observed that was most acute in the absence of A36 (Figs 2 and 3). This was due to impaired function of the F13 protein, because restoration of WT F13 repaired the plaque size defect (Fig. 4). The reduction in the plaque size of F13mCherry-expressing viruses was accompanied by a reduction in the number of virions reaching the cell surface (Fig. 5), suggesting a defect in either the formation or transport of IEVs. Electron microscopy revealed that F13mC caused aberrant wrapping of IMVs by intracellular membranes, and this defect was apparent in all viruses expressing F13mC, not just viruses lacking A36 (Figs 6 and 7).

F13 is essential for the wrapping process that results in the formation of IEVs [36], and drugs that target F13, such as IMCBH [47] and ST-246 [48], inhibit this wrapping process. The VACV B5 protein is also required for IEV wrapping [34, 35], although only its transmembrane domain and a small part of its lumenal domain are essential [49–51]. Viruses in which one or more of the B5 SCR domains are deleted produce IEVs that are transported to the cell surface but are released more easily, resulting in increased EEV [49, 52]. Herrera *et al.* [49] described IEVs (visualized by EM) with loosely attached envelopes that are similar to the bubble virions described here. However, these differ in that the B5 mutations disrupt the interaction between the inner and outer IEV envelopes, resulting in virions with additional lumenal material. In contrast, in the F13mC virus the outer and inner IEV envelopes remain tightly apposed (see Fig. 6) and it is the interaction between the inner IEV envelope and the IMV surface that is disrupted, resulting in virions carrying additional cytoplasmic material.

It is intriguing that the same wrapping defect found in all three GmC viruses described in this study leads to such different changes in plaque size. A similar situation was described in which the expression of a GFP-tagged full length B5 protein produced a defect that was only detectable in a virus lacking expression of A33 [53]. Both A36 and F13 are associated with the membranes that wrap around IMVs and are oriented into the cytoplasm. During wrapping, A36 is excluded from the space between the IMV and the inner wrapping membrane and accumulates on what becomes the outer IEV membrane [19]. It is possible that this exclusion occurs simply because A36 is being squeezed out as F13 interacts with the IMV surface. The defect in wrapping in the presence of F13mC might simply be steric, in that there is no room for F13mC to interact normally with the IMV surface and the zippering up of the interaction between the wrapping membranes and IMV is hindered by this bulky addition to F13. It is also possible that A36 is somehow involved in this process. Although A36 is not required for wrapping, its presence may somehow enhance the F13–IMV interaction. This could explain the higher prevalence of bubbles observed in the v∆A36-GmC virus, but cannot satisfactorily explain the severe plaque size defect.

Excluding A36, only F13 is located entirely within the cytoplasm, making it a good candidate to form the alternative link between IEVs and kinesin-1. Large protein tags such as mCherry can potentially interfere sterically with protein–protein interactions. If F13 was the alternative link between IEVs and kinesin-1, then anything that interfered with the establishment of this link might induce a larger defect when A36 is absent compared to when it is present, just as was observed for plaque size. However, both vGmC and v∆A36-GmC show a similar reduction (60–70 %, Fig. 5c) in the number of virions that reach the cell surface. Thus, the most plausible explanation for the severe plaque size defect of v∆A36-GmC is the contribution of A36-mediated actin tail formation in virus spread. Actin tail formation enhances the ability of virions to contact neighbouring cells [54], thus lowering the number of virions required at the cell surface to guarantee spread. Therefore, a reduction in virus egress will have a much larger impact on plaque size when actin tails are absent compared to when they are present. Furthermore, proteins A36 and A33, which are needed for actin tail formation, also induce actin tails underneath superinfecting virions, thereby enhancing virus spread further [55, 56]. These are the reasons why v∆F12-GmC (which has a functional A36) has far fewer virions reaching the cell surface than v∆A36-GmC but produces significantly larger plaques. Similarly, it was recently reported that disruption of F12 caused a larger plaque size defect when A36-mediated actin tail formation (but not A36-mediated kinesin-1 recruitment) was disrupted compared to when A36 was fully functional [42].

In summary, this paper reports that the addition of a fluorescent protein onto the C terminus of the F13 protein causes a defect in the wrapping of IMVs to form IEVs. The IEVs that are formed have less tightly attached outer membranes, larger volumes of cytoplasm between the IMV and IEV membranes, and often more than one IMV particle wrapped within the IEV membranes. This defect causes reduced transport of virions to the cell surface and reduced spread from cell to cell. Therefore, although the attachment of fluorescent proteins to F13 has been reported as a strategy to visualize IEV movement [9, 28, 40, 57, 58], it is important to recognize this could affect virus wrapping and transport. While the data presented here, which show that the fusion of mCherry to F13 results in a wrapping defect, should not automatically be extrapolated to other F13 fluorescent tagging strategies, it should be noted that EGFP and mCherry are very similar in size (239 versus 236 amino acids) and pI (5.58 versus 5.62), and have similar protein folds. Minor defects have been reported for some viruses expressing a GFP-tagged F13. Specifically, in a study by Geada *et al.* [9] a spreading defect is described that was most apparent in the VACV IHD-J strain. Therefore, while these viruses are useful tools for tracking VACV virions, they are imperfect reagents and so data should be interpreted cautiously.

## Methods

### Plasmids

A DNA fragment encompassing the F13 orf along with 376 bp of upstream sequence was amplified by PCR from VACV genomic DNA using oligonucleotides F13UPF1 (5′-caccgaattc cgtattggac atgcttatgt acgtag-3′) and F13R1 (5′-cttgtacagg atccgctagc ccaattttta acgatttact gtggctagat acc-3′). A fragment encompassing the F13 stop codon and 376 bp of downstream sequence was amplified with oligos F13DNF1 (5′-gctagcggat cctgtacaag taaaaaaaag aaaatagaga cgtatagaac gc-3′) and F13DNR1 (5′-caccaagctt ctccctgttc gtgggaacgc-3′). The two fragments were spliced together using splicing by overlap extension [59] to generate a fragment encoding the F13 orf with recognition sequences for the *Nhe*I, *Bam*HI and *Bsr*GI restriction enzymes introduced just before the F13 stop codon. This fragment was inserted into the *Eco*RI*-Hin*dIII restriction sites of pSJH7, a pUC13 plasmid expressing the *Escherichia* *coli* guanine phosphoribosyltransferase (*Ecogpt*) gene from the VACV 7.5K promoter [60] to produce pSJH7-F13. Subsequently the *Nhe*I-*Bsr*GI restriction fragment encoding the mCherry protein was subcloned from pUC13-EcoGPTmCherry [26] into the *Nhe*I-*Bsr*GI sites introduced before the F13 stop codon to generate plasmid pSJH7-F13mCherry (pF13mC).

### Cell culture and virus propagation

RK-13 cells (rabbit kidney cell line, ATCC CCL-37) were maintained in minimal essential medium (Gibco) supplemented with 10 % foetal bovine serum (FBS) and penicillin–streptomycin (Pen/Strep, Gibco). HeLa cells (human cervix adenocarcinoma, ATCC CCL-2) were maintained in minimal essential medium supplemented with 10 % FBS, non-essential amino acids and Pen/Strep (Gibco 11140). D98-OR, a hypoxanthine phosphoribosyl transferase-deficient HeLa cell-line [61], CV-1 (African green monkey cell line, ATCC CCL70) and BS-C-1 (African green monkey cell line, ATCC CCL-26) cell lines were maintained in Dulbecco's modified Eagle's medium (Gibco) supplemented with 10 % FBS and Pen/Strep.

All of the viruses used in this study were amplified in RK-13 cells and titrated by plaque assay on BS-C-1 cells using a carboxymethyl cellulose overlay. A recombinant virus in which the *F13L* gene has been disrupted by insertion of the *EcoGPT* gene was a kind gift from Bernard Moss (NIH, NIAID, Bethesda, Maryland, USA) [36]. VACV expressing GFP fused to the A5 capsid protein, WT (vA5GFP) [27], as well as viruses lacking expression of F12 (v∆F12-A5GFP), A36 (v∆A36-A5GFP) [23], or both (v∆A36∆F12-A5GFP) [42] have been described.

### Imaging double-fluorescent IEVs using infection/transfection

HeLa cells seeded into six-well plates were transfected with pF13mC using the Transit-LT1 transfection reagent (Mirus Bio LLC) and 16 h later were infected with vA5GFP at 2 p.f.u./cell. Infected cells were lysed in PBS with 1 % NP-40 and insoluble material was removed by centrifugation (15 000 ***g***, 15 min, 4 °C). Clarified lysates were analysed by SDS-PAGE and immunobloting using rat mAb 15B6 that recognises VACV protein F13 [6] and mouse mAb AB1.1 that recognises VACV protein D8 [14]. The blots were probed with IRDye-conjugated secondary antibodies (LI-COR) and imaged using a LI-COR Odyssey scanner.

To estimate whether IEVs could be labelled with F13mCherry, HeLa cells were seeded at low density in glass-bottomed tissue culture dishes (MatTek), and transfected and infected as described above. The cells were then imaged between 8 and 10 h p.i. using a Zeiss LSM780 confocal laser scanning microscopy system mounted on an AxioObserver.Z1 inverted microscope with a 64× Plan Apochromat objective (NA; 1.4) and Zen (Zeiss) acquisition software. Each cell was imaged for a total of 2 min at a rate of 0.5 frames s^−1^.

### Construction and characterization of recombinant viruses

VACV expressing F13mC were generated by transient dominant selection [62]. CV-1 cells were infected with vA5GFP, v∆F12-A5GFP or v∆A36-A5GFP at 0.1 p.f.u./cell and subsequently transfected with pF13mC using TransIT-LT1 (Mirus Bio LLC). The resulting viruses were selected by three rounds of plaque purification in the presence of mycophenolic acid, xanthine and hypoxanthine to select for EcoGPT expressing intermediate viruses that had incorporated the pF13mC plasmid. GFP+ve and mCherry+ve viruses were then plaque-purified three additional times without drug selection. PCR using oligos EcogptDCF1 (5′-cgtcacctgg gacatgttg-3′) and EcogptDCR1 (5′-gacgaatacg acgcccatat c-3′), producing a PCR product of 421 bp when the EcoGPT cassette is present, was used to identify isolates that had undergone a second recombination event, removing the EcoGPT cassette along with the WT F13 allele. PCR using primers F12ForDC1 (5′-atgttaaaca gggtacaaat cttgatga-3′) and F12RevDC1 (5′-catctttgat ctcgatggaa tgca-3′), giving a ~2-kbp fragment for a WT allele and a ~800-bp fragment for the ∆F12 allele, was used to confirm the identity of the F12 allele in each of the viruses. PCR using primers DCA36SeqF1 (5′-gcgtataacc tgtatacagc tgg-3′) and DCA36SeqR1 (5′-accgtttcat ccatctgtct attg-3′), giving a 1085-bp fragment for a WT A36 allele and a 658-bp fragment for the ∆A36 allele, was used to confirm the identity of the A36 allele in each of the viruses. This process failed to identify a A5GFP+ve, F13mCherry+ve, EcoGPT –ve v∆A36. For this virus, negative selection was used to enrich for recombinants that had lost the EcoGPT cassette. The intermediate v∆A36 virus was used to infect D98-OR cells at low m.o.i. in the presence of 6-thioguanine, which is toxic when EcoGPT is present. The resulting GFP+ve, mCherry+ve, EcoGPT –ve viruses were isolated by three additional rounds of plaque purification on BS-C-1 cells without drug selection. To generate a v∆A36-A5GFP revertant virus, the process described above was repeated, infecting with v∆A36-GmC and transfecting with pSJH7-F13.

Expression of F13mCherry by all recombinant viruses was assessed by SDS-PAGE and immunoblotting as described above. The plaque size produced by the various recombinant viruses was assessed by standard plaque assay on BS-C-1 cells. Briefly, monolayers of BS-C-1 cells in six-well plates were infected with 20–30 p.f.u./well. After 1 h of infection the inoculum was replaced with an overlay containing 1.5 % carboxymethyl cellulose. Cells were incubated at 37 °C for 2 to 9 days (until plaques could be visualized and were large enough to measure). Cells were fixed and stained with crystal violet solution (0.115 % crystal violet and 0.005 ammonium oxalate in 25 % v/v ethanol). Plaques were imaged using an AxioObserver.Z1 inverted microscope. Plaque surface area was measured using AxioVision (release 4.8) software. A minimum of 15 measurements from 3 independent wells (for a total of 45 measurements) were used to carry out statistical analysis using the statistical software package GraphPad Prism (version 5.04).

### Analysis of virion egress by surface B5 staining

To determine the efficiency of virus egress, the number of virions on the cell surface was measured by surface staining for the CEV/EEV surface protein B5 as described [23]. Briefly, sub-confluent BS-C-1 cells seeded in 24-well plates (for flow cytometry) or onto poly-d-lysine-coated coverslips in 12-well plates (for microscopy) were infected at 5 p.f.u./cell. At the time of sample collection, cells were placed on ice and the medium was replaced for 45 min with fresh pre-cooled medium (DMEM 2.5 % FBS) containing the rat mAb 19C2 [6] that recognizes the VACV B5 protein. The rat mAb 15B6 [6] that recognizes F13 was used as a negative control.

For analysis by flow cytometry, the medium was replaced by fresh pre-cooled medium containing a goat anti-rat IgG Alexa Fluor 647-conjugated secondary antibody (Life Technologies) for 45 min on ice. Subsequently, cells were dissociated using AccuEasy cell-lift solution (Biotium) and fixed with 4 % paraformaldehyde. Surface B5 staining levels were measured using a Becton Dickinson Cytek FACScan DxP8 and analysed using MoFlo software. Statistical analysis of triplicate samples was carried out using GraphPad Prism.

For manual counting of the number of surface virions by confocal microscopy, B5-stained infected cells on glass coverslips were fixed with 4 % paraformaldehyde in 250 mM HEPES pH 7.5 for 10 min on ice followed by 8 % paraformaldehyde (250 mM HEPES pH 7.5) for 20 min at room temperature. B5 staining was detected using a goat anti-rat IgG Alexa Fluor 546-conjugated secondary antibody (Life Technologies). Slides were mounted (10 % w/v Mowiol4–88 (CalBiochem), 25 % v/v glycerol, 100 mM Tris-HCl pH 8.5, 0.5 µg ml^−1^ DAPI (4',6-diamidino-2-phenylindole; Sigma) and imaged using a Zeiss LSM780 confocal laser scanning microscopy system mounted on an AxioObserver. Z1 inverted microscope using a 64× Plan Apochromat objective (NA; 1.4) and Zen (Zeiss, 2011 version) acquisition software. Z-stacks encompassing the full volume of the cell were acquired and used to generate maximum fluorescence intensity projections down the *z*-axis. Images were processed and analysed using Zen, ImageJ and Photoshop (Adobe) software.

### Electron microscopy

For the analysis of virus wrapping by EM sub-confluent, HeLa cells were infected at 5 p.f.u./cell. For labelling with a fluid phase marker, cells were incubated with 10 mg ml^−1^ horseradish peroxidase (HRP) dissolved in cell culture medium for 1 to 2 h prior to fixation. At 8–10 h p.i. cells were fixed in 0.5 % glutaraldehyde (200 mM sodium cacodylate pH 7.4) for 30 min and washed in 200 mM sodium cacodylate. For visualization of HRP, samples were reacted with a metal-enhanced DAB substrate kit (Thermo Scientific) for 30 min. All samples were postfixed (1 % osmium tetroxide, 1.5 % potassium ferrocyanide) for 60 min at room temperature. Cells were washed in water, incubated in 0.5 % magnesium-uranyl acetate overnight at 4 °C, washed again in water, dehydrated in ethanol and embedded flat in Epon. Sections were cut parallel to the surface of the dish and collected onto slot grids. Lead citrate was added for contrast. Images were acquired using a CCD camera mounted on a FEI Tecnai G2 transmission electron microscope.

## Supplementary Data

13936 Supplementary File 1

## Supplementary Data

11829 Supplementary File 2

## Supplementary Data

7427 Supplementary File 3

## Supplementary Data

9814 Supplementary File 4

## Supplementary Data

98 Supplementary File 5
