# Supplementary material for: Tagging of the vaccinia virus protein F13 with mCherry causes aberrant virion morphogenesis
Source: J Gen Virol. 2017 Sep 20;98(10):2543–55. doi: 10.1099/jgv.0.000917 (PMC5725974; doi:10.1099/jgv.0.000917)
Supplement: Supplementary File 5 [file jgv-98-2543-s005.pdf]

Supplemental material legends

Movie S1: HeLa cells were transfected with a plasmid expressing F13mC under the control of the F13 promoter and were infected 24 hours later with vA5GFP with 2 p.f.u./cell. Cells were imaged from 8 hpi for a period of 20 min. Movies have been speeded up 30-fold.

Movie S2: HeLa cells were transfected with a plasmid expressing F13mC under the control of the F13 promoter and were infected 24 hours later with vΔA36-A5GFP with 2 p.f.u./cell. Cells were imaged from 8 hpi for a period of 20 min. Movies have been speeded up 30-fold.

Movie S3: HeLa cells were transfected with a plasmid expressing F13mC under the control of the F13 promoter and were infected 24 hours later with vΔF12-A5GFP with 2 p.f.u./cell. Cells were imaged from 8 hpi for a period of 20 min. Movies have been speeded up 30-fold.

Movie S4: HeLa cells were transfected with a plasmid expressing F13mC under the control of the F13 promoter and were infected 24 hours later with vΔA36ΔF12-A5GFP with 2 p.f.u./cell. Cells were imaged from 8 hpi for a period of 20 min. Movies have been speeded up 30-fold.
